# The correlation between the gleason score of the biopsy and that of the prostatectomy patch

**DOI:** 10.1016/j.amsu.2021.02.015

**Published:** 2021-02-26

**Authors:** W. Bai, Y. Fadil, O. Idrissi, M. Dakir, A. Debbagh, R. Abouteib

**Affiliations:** a Service d'urologie, hôpital Ibn Rochd, CHU casablanca, Casablanca, Morocco; b Faculté de médecine et de pharmacie casablanca, université Hassan II, Casablanca, Morocco

## Abstract

Since the advent of massive dosage of prostate specific antigen (PSA), prostate cancer has become a major public health problem. It is currently the most common cancer and the second leading cause of cancer death in men (after lung cancer). More than 670,000 new cases are diagnosed annually worldwide.

This is a retrospective study including all patients treated for prostate cancer by radical prostatectomy at the Ibn Rochde University Hospital in Casablanca between January 2017 and December 2020, i.e. a period of 4 years. At the end of our study, we identified 18 cases of radical prostatectomy.

## Highlights

• The aim of this study is to assess the correlation between the biopsy Gleason score and that of the radical prostatectomy specimen.
• This will allow the reliability of this biopsy histopronostic factor to be assessed in predicting Gleason scores for surgical specimens.

## 1 Introduction

Since the advent of massive dosage of prostate specific antigen (PSA), prostate cancer has become a major public health problem. It is currently the most common cancer and the second leading cause of cancer death in men (after lung cancer). More than 670,000 new cases are diagnosed annually worldwide [1].

For the assessment of the histological prognosis, many classification systems have been proposed, but in the opinion of several authors, the Gleason score is the most important among the predictors of survival [2]. It is obtained by adding the values of the two most represented contingents in the sample [3]. Unfortunately, this Gleason score on a biopsy is sometimes different from that on a surgical specimen, making it difficult to assess the degree of aggressiveness.

From the comparison between the biopsy Gleason scores and the Gleason scores of the prostatectomy specimens, we determined the number and percentage of patients who had an overestimation or an underestimation of the Gleason score and those for whom the score has not changed.

The aim of this study is to assess the correlation between the biopsy Gleason score and that of the radical prostatectomy specimen. This will allow the reliability of this biopsy histopronostic factor to be assessed in predicting Gleason scores for surgical specimens.

## 2 Material and methods

This is a retrospective study including all patients treated for prostate cancer by radical prostatectomy at the Ibn Rochde University Hospital in Casablanca between January 2017 and December 2020, i.e. a period of 4 years. At the end of our study, we identified 18 cases of radical prostatectomy.

Were included in this study, all cases of prostate biopsies and surgical specimens for which the pathological report included a diagnosis of cancer with a Gleason score. Cases which only had the result of biopsies or surgical specimens, as well as cancer results from less than six biopsy fragments were excluded from the study. We then analyzed the correlation between the Gleason score of the biopsies and that of the surgical specimen for each patient, then between the three histological groups of tumor differentiation: moderately differentiated cancer (score 5–7), poorly differentiated cancer (score 8–10).

An operating sheet enabled us to collect the following data: age, sex, history, risk factors, symptoms, paraclinical examinations, and anatomopathological results of the prostate biopsy and the prostatectomy part (number of cores, number of positive biopsy, biopsy length, cancer length, histological type, Gleason score, extra capsular extension, perinervous infiltration, vascular invasion, presence of high grade pine, presence of ASAP, IHC study.

The analysis was done in the laboratory of epidemiology of the faculty of medicine and pharmacy of casablanca and the software which been used is SPSS version 20.

All data were included in the Excel spreadsheet, the comparative study of the data was done by Student's t-test. The qualitative variables were compared by the chi2 test. The results were considered statistically significant for a p < 0.05.

Sensitivity (SE), specificity (SP), positive predictive value (PPV) and value negative predictive force (NPV) were calculated for group Gleason biopsies well differentiated and moderately differentiated. In addition, the poorly differentiated group was excluded from this calculation due to the small number of patients classified in this group (1To determine the accuracy of each study, we compared the biopsies and prostatectomy specimens using the statistical agreement test of Kappa proposed by Landis and Koch

work was reported according to STROCSS criteria [20].

registration unique identifying number esearchregistry6499 https://www.researchregistry.com/browse-the-registry#home/

## 3 Results

A-the biopsy Gleason score:

For the distribution of patients according to their Gleason score, the results were as follows: 78% of patients had a score between 5 and 7 and 22% of the sample studied had a score between 8 and 10(Table 1).

B-The correlation between the biopsy Gleason score and the gleason score of radical prostatectomy:

**Table 1:** Distribution of patients according to the Gleason score of the biopsy and that of the prostatectomy specimen.

	Biopsies	Pièces opératoires
Score 5-7	14 (78%)	11 (61%)
Score 8-10	4 (22%)	7 (37%)

Regarding the differentiation groups (Table 1), we noted. - Moderately differentiated group (Gleason score of 5–7): 14 patients belonged to this group at the biopsy. 11 of these patients (78%) remained in the same group at the prostatectomy patch, on the other hand 3 patients (22%) were under-staged and moved to the poorly differentiated group - Poorly differentiated group (Gleason score 8 to 10): 4 patients were classified in this group on biopsy. All of these patients (100%) remained in the same group at the prostatectomy room.

**Table 2:** Distribution of patients according to differentiation groups on biopsy and prostatectomy specimen.

Score de Gleason sur la biopsie	Score de Gleason sur la pièce opératoire		
	Effectif qui ne change pas de groupe	Effectif qui change de groupe	Total
5–7	**11**	**3**	**14**
8–10	**4**	**0**	**4**
total	**15 (84%)**	**3 (16%)**	**18**

Agreement was 78% in the moderately differentiated group and 100% in the poorly differentiated group.

**Fig. 1:**
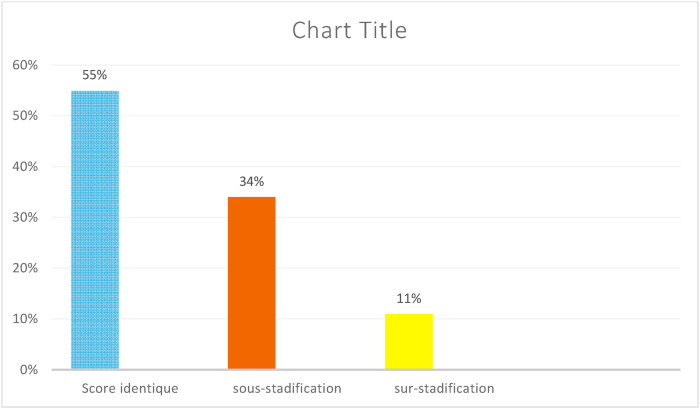
Concordance between the biopsy Gleason score and that of the prostatectomy specimen.

For the Gleason score There was a concordance in 55% of the cases. The Gleason score was lower than that of the piece (under-staging) in 34%. Moreover, it was higher than that of the piece (on -staging) in only 11% (Fig. 1).

Agreement was 78% in the moderately differentiated group and 100% in the poorly differentiated group.

**Fig. 2:**
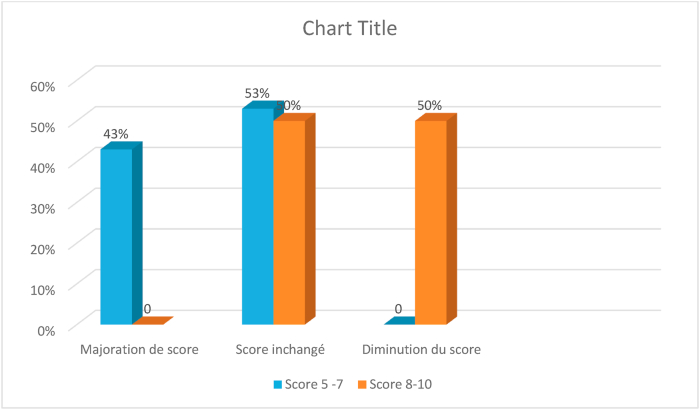
Increase or decrease in the gleason score on the biopsy depending on the group.

The increased score occurred in 43% of biopsies in the moderately differentiated group (see Table 2). The decrease in score occurred in 50% of biopsies in the poorly differentiated group (Fig. 2) and (Table 3).

**Table 3:** Comparison of biopsy Gleason scores and specimens radical prostatectomy.

Effectif	Score de gleason sur pièce opératoire	Total
Score	6 7 8 9 10	
6	2 3 0 0 0	5
De		
Gleason 7	0 6 3 0 0	9
biopsique		
8	0 1 2 0 0	3
9	0 0 1 0 0	1
10	0 0 0 0 0	0
Total	3 9 6 0 0	18

## 4 Discussion

The Gleason score of the radical prostatectomy patch is an important prognostic factor in patients with prostate cancer. However, the therapeutic algorithm based partially on the biopsy grade depends largely on the correlation between the histology of the biopsy and that of the radical prostatectomy piece. This study was designed to assess the relationship between the Gleason score of the biopsy and that of the operative specimen.

The results of our study demonstrated that although there was a good agreement, several difficulties are faced when the treatment decision is guided by the biopsy Gleason score.

In our series like Prost's, the score difference of one point was greater than that of at least two points [4].

The concordance in our series was 55%, an under-staging of 34% and an over-staging of 11%. The worst correlation noted was that of the well-differentiated group with a concordance of 78% and an under-staging of 43%.

### 4.1 Indeed, similar results have been previously described in the literature

However, other studies have reported different results:

In England, Bott et al., found in a series of 628 patients a concordance of 60%, an under-staging of 29%, an over-staging of 11% and a kappa concordance coefficient of 0.40 [5].

Lattouf et al. In a larger series of 393 patients, they showed a 69% agreement with 21% under-staging, 10% over-staging and a kappa coefficient of 0.30 [6].

Mian et al. In a series of 426 patients, showed a correlation of 67% with 25% under-staging, 8% over-staging and a kappa coefficient of 0.43 [7].

A study by San Francisco et al., in a series of 466 patients, found a concordance of 74%, under-staging in 17% of cases, over-staging in 9% of cases and a kappa coefficient of 0.43 [8].

Chun et al. Reported, in a series of 4789 patients, a correlation of 66% with 6% under-staging, 28% over-staging and a kappa concordance coefficient of 0.60 [9].

In Japan, Tomioka et al. observed in a series of 223 patients a concordance of 61%, an under-staging of 21%, an over-staging of 17% and a kappa coefficient of 0.37 [10].

The perfect correlation between the biopsy Gleason score and that of the operative specimen in our work was almost identical to that of Bott and Tanioka, and less than that of Tomioka et al.

There are many reasons why the Gleason score varies between the biopsy and the prostatectomy patch:

First, the quantity taken by the biopsy is small.

20 mm taken by an 18 gauge needle is only 0.04% of the volume of a 40 ml prostate. Boston reported that the Gleason score on the prostate biopsy with an 18 gauge needle performed in 316 patients was the same as in the exhibit prostatectomy only in 35% of cases [11]. The literature review also shows 2 distinct periods. The first, which corresponds to the use of 14 gauge needles, is characterized in various series by a satisfactory agreement between the two Gleason scores. The second period, that of the use of 18 gauge needles for ultrasound-guided biopsies, is distinguished by the reduction in the volume of the sample by 50%, which explains a poorer match. However, there is no correlation between the error in the biopsy score and the size of the prostate, its weight, the PSA level [11,12] or the number of carrots. Saturation biopsy models were evaluated to see if Gleason's concordance between the biopsy and the prostatectomy patch could be improved with increasing carrot numbers. The largest study [9], including 4789 patients, found no difference in the accuracy of Gleason for patient staging based on the number of cores at biopsy (≤10 biopsies).

Another explanation for the increased grade is the maturation of the cancer in the preoperative period from biopsy to the time of surgical excision. However, Freedland et al. [13] showed that the delay between biopsy and surgery was not associated with an increase in the grade of the disease. In contrast, the significant decrease in the grade of the prostatectomy specimen biopsy is difficult to conceptualize. Does a high grade tumor naturally turn into a low grade tumor?, there is a rare possibility that the biopsy excised the high-grade cancer, leaving the patient with only the low-grade disease.

Another plausible explanation for the increase or decrease in grade concerns the “tertiary” mark of the Gleason. A tertiary motif representing less than 5% of the tissue may be present, but not included in the standard Gleason score. Some have proposed that if a tertiary motif of 4 or 5 is present, it should be reported as a tertiary number on the biopsy Gleason score, even when it represents less than 5% of the tumor [14]. The same authors have found that this higher number has prognostic significance to correlate with high stage disease, especially in low grade tumors.

However, in our series we did not record any tertiary Gleason grade. A pattern superior to the biopsy may turn out to be a primary or secondary pattern on the prostatectomy patch and therefore may count towards the increased guard. Alternatively, the concept of higher Gleason grade may also explain the decrease in grade. Current biopsy reports provide the Gleason score and percentage of tumor invasion for each biopsy core, which can lead to multiple scores for a patient. by Gleason.

When analyzing the results according to the differentiation group, the lack of agreement observed between the biopsy Gleason score and that of the operative specimen may be the consequence of a change of score group. In fact, the change in the Gleason score between the biopsies and the operative specimen can move the same patient into a higher or lower score group. Nevertheless, an underestimation by biopsies of the score of two points or more should not have an impact on the treatment protocol, provided that the patient does not change the score group. On the other hand, in some cases, the change of only one point of the Gleason score can change the score group and therefore modify the treatment protocol. Classifying patients into three distinct groups (well-differentiated, moderately and poorly differentiated tumor) allows for greater amplitude in the prediction of Gleason and is consistent with clinical interpretation. This classification improves the prediction of the definitive Gleason score by prostate biopsies [4,15,16]. With this differentiation criterion, the degree of precision was reduced from 37% to 72% in the Prost study [4].

Finally, the reproducibility of the Gleason score is poor during iterative analyzes by the same or by other pathologists. It can be improved by pathological examination centralized in a tertiary center [4,12]. Gleason himself reports an intra-observer reproducibility of only 50% in the case of re-reading of the slides. Inter-observer reproducibility varies from 22% to 37% [17,18]. Fernandes has shown that a well-differentiated tumor on prostate biopsy is a weak predictor of a well-differentiated tumor or of a tumor localized to the prostate after radical prostatectomy. On the other hand, cancer with a high Gleason score on prostate biopsy is most often associated with extra-prostatic disease and with a poorly differentiated tumor on the radical prostatectomy part [19].

Currently, several local therapy options exist for prostate cancer (Radiotherapy, Brachytherapy and Ablatherm). We believe that the inaccuracy of the Gleason score in predicting the Gleason of the prostatectomy specimen makes the choice between the multiple forms of radiation therapy rather arbitrary.

## 5 Conclusion

The Gleason score is the best histopronostic criterion in prostate cancer. Unfortunately, the reproducibility of the Gleason score was poor in iterative analyzes by the same or by other pathologists. Furthermore, prostate cancer is heterogeneous and biopsies do not accurately reflect the actual tumor architecture. In practice, the classification of patients according to the three distinct groups of tumor differentiation could increase the correlation between the biopsy Gleason score and that of the operative specimen.

The evaluation of the latter on prostate biopsies is a determining element in the discussion of treatment options. This assessment does not allow predict the course of prostate cancer. This classification system has limitations significant to accurately predict the Gleason score of the

radical prostatectomy. Of course, our study has limits which are: the size of the sample and the heterogeneity of patients and pathologists. However, our results, similar to those in the literature, suggest that the Gleason score of the biopsy does not imperfectly reflects that of the operative part.

In the hope that future research will improve the techniques of currently recognized staging, physicians and patients should take awareness of our final assessment of the biopsy Gleason score to be predicted the aggressiveness of the prostate tumor for the purpose of better management.

## Sources of funding

No Sources of funding

Provenance and peer review.

Not commissioned, externally peer-reviewed.

## Declaration of competing interest

No Conflicts of interest.
